# Effect of High-Salt Diet on Memory and Behavior in Mice Expressing Human Apolipoprotein Epsilon-4 (APOE4) Allele

**DOI:** 10.3390/neurosci7020043

**Published:** 2026-04-07

**Authors:** Riad Abdulmoniem, Mia Rivers, Gabriel Carter, Syed J. Khundmiri, Jahn N. O’Neil

**Affiliations:** Department of Physiology and Biophysics, Howard University College of Medicine, Washington, DC 20059, USA; riadha2010@hotmail.com (R.A.); carterg@alumni.vcu.edu (G.C.)

**Keywords:** APOE3, APOE4, dietary salt, memory, behavior

## Abstract

Apolipoprotein epsilon (APOE) is a small molecular protein that regulates lipid and lipoprotein homeostasis. Several reports demonstrated that apolipoprotein epsilon-4 allele (APOE4) expression significantly increases the genetic risk of Alzheimer’s disease (AD) and chronic kidney disease. However, there is inconsistent evidence of the association of AD with dietary habits, especially salt intake. Therefore, we hypothesized that high dietary salt intake would exacerbate cognitive decline in mice expressing the human APOE4 allele. We used human APOE (APOE4 and APOE3) knock-in mice to test this hypothesis. Young adult male and female mice aged 5–7 months old (*n* = 18 in each group) were fed a 4% NaCl (high-salt) or a 0.1% NaCl (low-salt) diet for 4 weeks. Metabolic cage studies were used to assess 24 h measurements of food and water intake, and urine output. Spatial memory and learning were determined using the Barnes maze test. Both the APOE3 and APOE4 mice on a low-salt diet had significantly decreased urinary volume, and female mice had lower body weight. The APOE4 mice on the low-salt diet (0.1%) performed significantly better on the 72 h probe test as compared to the APOE4 mice on 4% salt diet. The results demonstrate an association among dietary salt, memory, and APOE4 genotype.

## 1. Introduction

Alzheimer’s disease (AD) is the most common neurodegenerative disorder and the leading cause of dementia worldwide. It is characterized by progressive cognitive decline, synaptic dysfunction, and neuroinflammation. Increasing evidence suggests that vascular dysfunction, hypertension, and chronic inflammation contribute to AD pathogenesis. Apolipoprotein epsilon (APOE) is a small molecular weight protein mostly produced in the liver, with smaller amounts locally produced in the brain, kidneys, spleen, adrenal glands, and macrophages [1]. APOE functions as a ligand for receptor-mediated clearance of chylomicrons and plays a critical role in reverse cholesterol transport [2]. In humans, three variants of the APOE allele are expressed, viz., APOE2, APOE3, and APOE4 [3]. The APOE3 allele, which expresses a cysteine at position 112 and an arginine at position 158, is the most common and is considered the control wild-type allele [4]. The APOE2 allele, which expresses cysteines at positions 112 and 158, is the least common and is defective in interaction with the lipoprotein receptor [5]. Both APOE2 and APOE3 preferentially bind small high-density lipoproteins (HDLs) [6]. The APOE4 allele, which expresses arginine at the 112 and 158 positions, binds to large triglyceride-rich very low-density lipoproteins (VLDLs) [7]. APOE gene polymorphisms are associated with the concentration of APOE present in circulation. The APOE2 allele carriers express the highest amounts of apolipoproteins, while the APOE4 carriers have the lowest concentration in serum [4]. The risk of cardiovascular (CVD) and chronic kidney disease (CKD) is inversely proportional to the concentration of circulating apolipoproteins, suggesting that carriers of the APOE4 allele are at risk of developing CVD and CKD [1,5,8]. The APOE4 allele is also implicated in the development of Alzheimer’s disease (AD). Having at least one APOE4 gene increases the risk of developing AD by 2-3-fold. A carrier of two APOE4 genes has an increased risk of association by approximately 8-12-fold [9,10]. AD is a progressive neurodegenerative disorder characterized by cognitive and functional decline that significantly impairs the quality of life. It is the most common age-related neurodegenerative disease and the primary cause of dementia [11].

Hypertension (HT), characterized by elevated blood pressure, has been increasingly recognized as a risk factor for the development of neurodegenerative dementias, including AD [12]. Approximately 50% of adults over the age of 60 have hypertension [13], and more than 10% over the age of 65 will develop AD [14]. About 75% of hypertensive patients over the age of 65 are salt-sensitive [15,16,17]. High dietary salt increases blood pressure in these individuals. However, the relationship between high dietary salt, expression of the APOE4 allele, and memory loss has not been established. Recent data suggest that dietary changes, such as the Mediterranean diet, the DASH (Dietary Approaches to Stop Hypertension) diet, and the MIND (Mediterranean–DASH Intervention for Neurodegenerative Delay) diet, may protect and/or delay AD [14]. Our laboratory has demonstrated that high dietary salt increases blood pressure in two models of aging, viz., Fisher Brown Norway (FBN) rats and C57Bl/6J mice [18].

Based on data from our laboratory and others showing that high dietary salt increases blood pressure, we hypothesize that an increase in dietary salt intake will cause premature memory loss and behavioral deficiency in adult mice expressing the APOE4 allele. The goals of this study were to determine if there is a direct link between dietary salt and memory loss in adult mice, with targeted expression of the human APOE4 allele in the brain. Our data, for the first time, demonstrated that reducing dietary sodium improved memory in mice carrying the APOE4 allele, while increasing dietary salt led to a significant decline in memory.

## 2. Materials and Methods

### 2.1. Animals

**Figure 1:**
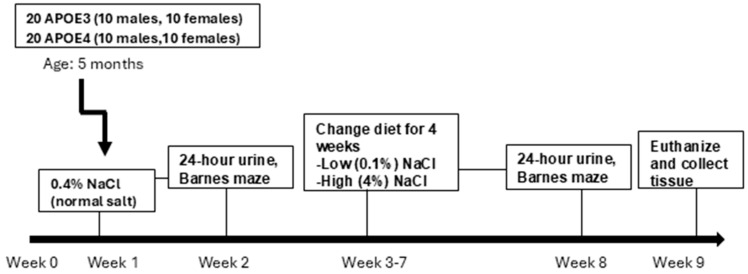
Animal demographics and experimental design. Male and female humanized APOE3 (strain 029018; *n* = 10 males and 10 females) and APOE4 (strain 027894; *n* = 10 males and 10 females) knock-in mice (5–7 months old) were initially maintained on a normal-salt diet (0.4% NaCl) to establish baseline metabolic and behavioral parameters (Week 2). Mice were then randomly assigned to either a low-salt (0.1% NaCl) or high-salt (4% NaCl) diet for 4 weeks, followed by metabolic and behavioral assessments (Week 8).

All animal experiments were performed according to the US Guide for the Care and Use of Laboratory Animals and approved by the Institutional Animal Care and Use Committee (IACUC) at Howard University (IACUC-MED-21-05). Male and female APOE3 expressing (Strain 029018) and male and female APOE4 expressing (Strain 027894) mice were purchased from Jackson Laboratories (Bar Harbor, ME, USA 04609). The mice were used in the experiment in the age range of 5–7 months. The mice were maintained in a temperature- and humidity-controlled vivarium at HU College of Medicine, provided food and water ad libitum, and subjected to a standard 12 h light–dark cycle. Behavioral manipulations were performed during the light phase. Figure 1 shows the animal demographics.

### 2.2. Treatment with Dietary Salt (NaCl)

The mice were first fed a normal-salt diet (0.4% NaCl) and underwent metabolic and behavioral tests to determine the effects of normal dietary salt. Following the control salt measurements, the animals were randomly divided into a low-salt (0.1% NaCl) and a high-salt (4% NaCl) group and were fed a diet containing 0.1% or 4% dietary salt (Figure 1). The experimental protocol was developed such that each normal-salt diet mouse served as its own control under different dietary salt conditions. During the treatment time, 1 male and 1 female APOE3 mice on a 0.1% low-salt diet, 2 female APOE4 mice on 0.1% low-salt diet, and 2 male APOE4 mice on a 4% high-salt diet died. For clarity, all figure legends explicitly indicate the number of animals analyzed per condition, the experimental groups compared, and the statistical tests used, enabling interpretation of each figure independent of the main text.

### 2.3. Urine Collection, Food and Water Intake

The mice were trained in metabolic cages (Catalogue No. 3M12D100; Braintree Scientific, Inc., Braintree, MA, USA) to collect 24 h urine volume along with food and water intake for 4–6 h on alternate days over three days. After training, the mice were housed in metabolic cages for 24 h. Body weight, food, and water intake were measured during the normal-salt diet condition and after the dietary changes. Urine collection was performed to assess physiological responses to dietary salt manipulation, including changes in urinary output and potential alterations in sodium and fluid balance. Urine was collected, measured, centrifuged at 5000× *g*, and stored in a −80 °C freezer until use.

### 2.4. Barnes Maze Apparatus and Testing Conditions

The Barnes maze behavioral circular apparatus (San Diego Instruments (SDI), San Diego, CA, USA) was constructed of white ABS plastic and contained 20 circular holes (2-inch diameter), evenly spaced along the perimeter of the maze. Only the target hole was deep enough for an animal to enter and led to an escape chamber (4 × 2 × 2 inches) beneath the maze floor. We used a 78-dB blow-dryer simulation, overhead lighting, and visual cues on the walls to drive escape behavior. Behavioral data, including latency, distance traveled, and hole investigations (nose pokes), were quantified using ANY-maze automated video tracking software version 7.33, which detects animal position and predefined zone interactions in real time, minimizing observer bias. The APOE3 or APOE4 knock-in mice completed three phases of Barnes maze testing: habituation, spatial acquisition, and a 72 h probe, as described by Sepulveda et al. [19], at normal (0.4% salt), low (0.1%), and high (4%) dietary salt. The Barnes maze test was conducted at 10 AM for all experiments and was blinded to the experimenter for the salt treatment.

#### 2.4.1. Habituation Phase

Animals were habituated to the Barnes maze testing conditions and allowed to freely explore the apparatus for 120 s before being gently guided to the target hole, where they remained for an additional 120 s.

#### 2.4.2. Spatial Acquisition (Learning Phase)

The mice learned over 4 days (four trials/training day (TD)) to use distal visual cues to locate the target hole and escape. At the beginning of the trial, the animals were placed under a start box in the center of the maze for 10 s before the start box was removed. A trial was terminated when the mouse entered the target hole or when 180 s had elapsed (trial completed). Consistent with standard Barnes maze protocols used in behavioral neuroscience studies, this duration provides sufficient time for animals to locate the target hole while minimizing fatigue and non-goal-directed exploratory behavior that may confound interpretation of spatial learning. The position of the target hole was randomly assigned and differed between the habituation and spatial acquisition phases. Latency (sec) to escape the maze by entering the target hole was calculated to assess spatial learning, while total distance traveled (m) was calculated to detect differences in locomotor activity. The number of hole investigations (total nose pokes) was automatically quantified using ANY-maze software version 7.33 as an indicator of exploratory behavior.

#### 2.4.3. Probe (Memory Phase)

At 72 h after the last training trial, the target hole was closed, and spatial learning and memory were assessed in a 90 s probe trial by measuring the latency to the closed target hole (primary latency), distance traveled, number of investigations of false holes before reaching the target hole (primary errors), and total nose pokes.

### 2.5. Immunohistochemistry

Ionized calcium-binding adaptor molecule 1 (Iba1) immunohistochemistry: Iba1 expression was determined using an antibody against Iba1 (polyclonal, Chemicon International, Temecula, CA, USA), a microglia marker. Iba1 is upregulated during activation and is a marker for neuroinflammation. Mice were perfused with ice-cold 0.1 M phosphate-buffered saline (PBS). Brains were removed, and the left side was post-fixed with 4% paraformaldehyde, followed 24 h later with placement in 30% sucrose for cryoprotection. The left brain was sectioned at 40 microns, and for visualization of microglia, a total of 8–10 systematic random sections through the hippocampus per brain were collected in 12-well plates. The sections were washed in 0.1 M PBS, incubated in 1% hydrogen peroxide for 30 min at room temperature (RT), washed again in 0.1 M PBS, and placed in 0.3% Triton X-100 for 10 min at RT. The sections were washed in 0.1 M PBS, then non-specific binding was blocked with 5% normal goat serum in 0.1 M PBS for 30 min at RT. The sections were incubated overnight in rabbit anti-Iba1 antibody diluted to 1:1000 in 0.1 M PBS containing 2% normal goat serum and 0.3% Triton X-100 at 4 °C. After incubation, the sections were washed in 0.1 M PBS and incubated in biotinylated secondary anti-rabbit antibody (cat. # BA-1000, Vector Laboratories, Burlingame, CA, USA) in 0.1 M PBS containing 2% normal goat serum for 90 min at RT. The sections were washed in 0.1 M PBS and incubated for 90 min in an ABC solution from the Vectorstain Kit (Vector Laboratories, Burlingame, CA, USA) at RT. The sections were rinsed in 0.1 M PBS and colorized using a DAB substrate kit (cat. # SK-4100, Vector Laboratories, Burlingame, CA, USA) for 6–10 min. All Iba1-immunostained sections were lightly counterstained in a 0.1% solution of cresyl violet, rinsed, dehydrated through an ascending graded series of alcohol, cleared in histoclear, and coverslipped with DPX.

### 2.6. Statistics

Experimenters conducting molecular studies and statistical analysis were blinded to the animal treatments. G*Power version 3.9.1.7 was used to determine the sample size needed to detect an effect of a given size with a given degree of confidence. The sample size was *n* = 4 per group. Organizing and populating indices were carried out in Microsoft Excel 365 (Redmond, WA, USA; Supplementary raw datasheet). The data were then exported to Prism 10 (GraphPad, San Diego, CA, USA). The data were subjected to a Shapiro–Wilk normality test and Levene’s test for homogeneity of variance. Differences between means were assessed by *t*-test, ANOVA, and GLM with repeated measures, followed by Tukey’s HSD or Bonferroni post hoc corrections to detect statistical differences among groups. All data are expressed as the mean ± SE, with significance set at *p* < 0.05.

## 3. Results

### 3.1. Metabolic Cage Studies

**Figure 2:**
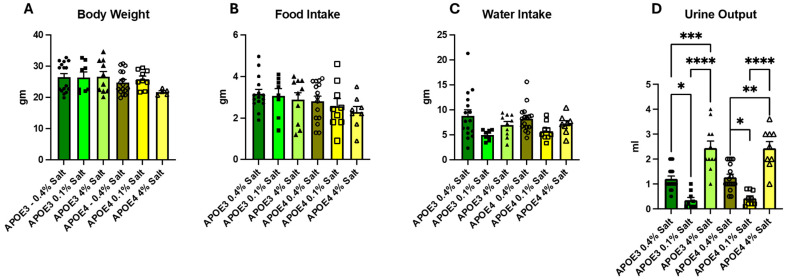
Effect of dietary salt on body parameters: body weight (**A**), food intake (**B**), water intake (**C**), and urinary volume (**D**) in APOE3 and APOE4 mice on normal salt (0.4% NaCl), low salt (0.1% NaCl) and high salt (4% NaCl). Each bar represents data as mean ± SE, *n* = 7 in low-salt treatment groups and *n* = 9 in high-salt treatment groups. Significance was calculated by Two-way ANOVA, followed by Bonferroni multiple comparison tests (* *p* < 0.05, ** *p* < 0.01, *** *p* < 0.001, and **** *p* < 0.0001).

To determine the effects of dietary salt on body weight, food and water intake, and urinary volume, the mice were trained on metabolic cages, as described in the Methods Section. Following training, the mice were housed in metabolic cages for 24 h, and urine was collected. At baseline on a normal-salt (0.4% NaCl) diet and after changes to low- (0.1% NaCl) or high-salt (4% NaCl) diet, the male mice had significantly higher body weight as compared to females in both the APOE3 and APOE4 carriers (Figure 2A and Supplementary Figure S1). However, the baseline body weight was not different between the groups. There was no significant difference in food and water consumption (Figure 2B,C and Supplementary Figure S2A). Urinary volume was significantly less in both the APOE3 and APOE4 mice on 0.1% dietary salt, as compared to the normal and 4% salt groups. (Figure 2D and Supplementary Figure S2B).

### 3.2. Effect of Salt on Memory in APOE3 and APOE4 Mice

**Figure 3:**
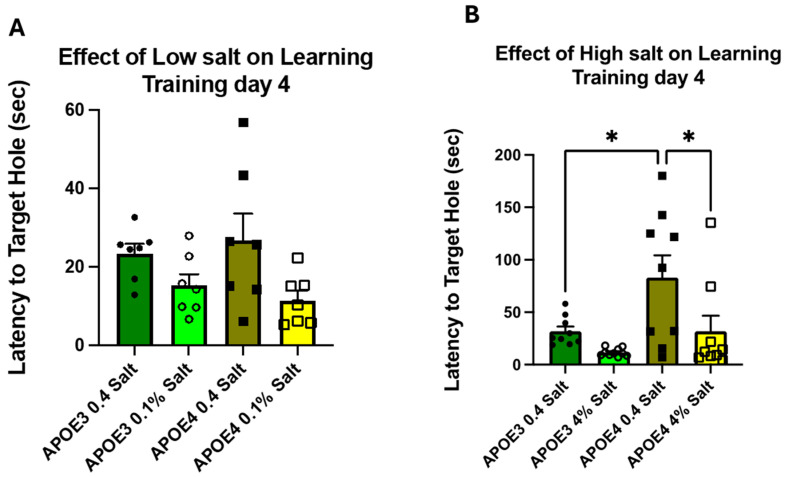
Memory and learning: the male and female APOE3 and APOE4 mice were trained, and their memory and learning abilities were determined by the Barnes maze test. The bars represent latency to target hole (sec) data as the mean ± SE on normal-salt (0.4%), low-salt (0.1% NaCl) (**A**) and high-salt (4% NaCl) (**B**) diets, *n* = 7 in the low-salt treatment groups and *n* = 9 in the high-salt treatment groups. No sex differences were observed. Significance was calculated by Two-way ANOVA, followed by Bonferroni multiple comparison tests (* *p* < 0.05).

During the four training days (TDs), the time required to escape the maze (primary latency) by entering the target hole was collected for all animal groups (Supplementary Figure S3). At baseline (week 2), on training day 4, there were no significant differences in primary latency (Supplementary Figure S3), primary errors, number of nose pokes, or distance traveled (Supplementary Figure S4). There were no significant differences between the sexes (Supplementary Figure S5); therefore, the data from the male and female mice were pooled in subsequent experiments. In week 8, following different salt diets, on training day 4, primary latency was significantly different in the APOE4 mice fed a normal-salt diet as compared to the APOE3 mice on an adapted salt diet. The APOE4 mice on a high-salt (4%) diet showed a significant change in primary latency compared to those on a normal-salt diet. There were no significant differences in any groups on a low-salt (0.1% salt) diet as compared to the normal-salt diet fed mice (Figure 3).

### 3.3. Memory Test Using 72 H Probe

**Figure 4:**
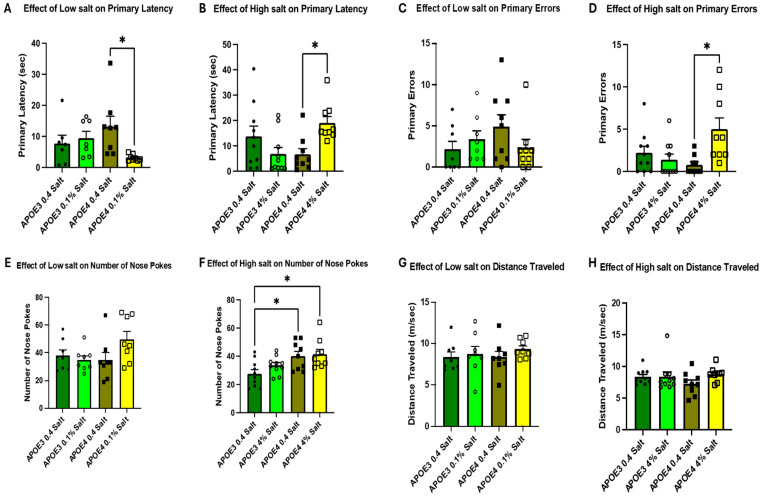
Memory in APOE3 and APOE4: 72 h after the 4th training day, memory to reach the target hole was measured by the Barnes maze test. Primary latency (**A**,**B**), primary errors (**C**,**D**), total nose pokes (**E**,**F**), and distance traveled (**G**,**H**) were measured. Each bar represents data as the mean ± SE, and *n* = 7 in the low-salt treatment groups and *n* = 9 in the high-salt treatment groups. No sex differences were observed. Significance was calculated by Two-way ANOVA, followed by Bonferroni multiple comparison tests. (* *p* < 0.05).

Following the completion of the training regimen, all mice completed a spatial memory probe 72 h after the last training day (TD4). Mice with the APOE4 allele on a low-salt (0.1% salt) diet showed significantly better primary latency compared to normal salt-fed mice (Figure 4A), as measured by the 72 h probe. In contrast, the APOE4 mice on 4% salt diet had significantly increased primary latency as compared to the normal salt-fed mice (Figure 4B). There were no significant effects of salt on memory recall compared to the normal-salt (0.4% salt) diet-fed APOE3 mice, as measured by the 72 h probe (Figure 4A,B). Similar to primary latency, the APOE4 mice fed a high-salt (4% salt) diet exhibited increased primary errors (Figure 4D) and number of nose pokes (Figure 4F), as compared to mice fed a normal-salt (0.4% NaCl) diet. Dietary salt did not affect the distance traveled in both genotypes (Figure 4G,H). The male and female data were combined as there were no significant differences between sexes (Supplementary Figure S4). The absence of significant differences during training, but the presence of differences during the probe test, suggests that dietary salt primarily affects memory retention rather than the acquisition of spatial learning.

### 3.4. Effect of Dietary Salt on Ionized Calcium-Binding Adapter Molecule 1 (Iba1) Expressing Microglial Cells

**Figure 5:**
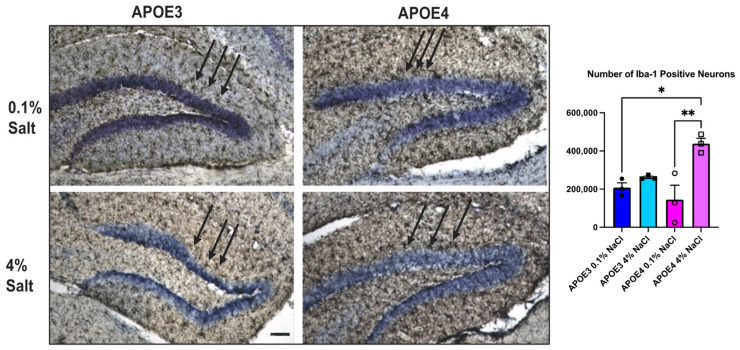
The effect of dietary salt on ionized calcium-binding protein (Iba-1) expression in the hippocampal formation: Following the 30-day feeding with a low (0.1% salt) or high (4% salt) diet, Iba-1 expression was measured by IHC, as described in the methods. A representative immunohistochemical (IHC, 10×) photomicrograph of the dentate gyrus, part of the hippocampal formation, is shown (*n* = 3 in each group). Scale bar = 100 μm. Significance was calculated by Two-way ANOVA, followed by Bonferroni multiple comparison tests. (* *p* < 0.05; ** *p* < 0.01). The arrows indicate representative Iba-1–positive microglial cells.

To determine whether dietary salt mediates changes in spatial learning and memory in the APOE4 mice through neuroinflammation, we examined Iba1-expressing microglial cells in the hippocampal formation. The hippocampal formation consists of the hippocampus, dentate gyrus, and subiculum. It plays a role in learning and memory. Immunohistochemistry of brain tissues revealed an increase in Iba1-positive microglial cells in the APOE4 mice compared to the APOE3 mice. The APOE4 mice on a high-salt (4% NaCl) diet exhibited a significantly higher number of Iba1-positive glial cells compared to the APOE3 mice on a high-salt (4% NaCl) diet and the APOE4 mice on a low-salt (0.1% NaCl) diet (Figure 5).

## 4. Discussion

The current understanding is that hypertension contributes to brain dysfunction and exacerbates the pathogenesis of AD [8,9]. This is likely due to compromised impairment of the cerebral microvasculature [10,11]. Expression of the APOE4 allele is a very well-known risk factor in the development of AD [20], and high dietary salt is known to increase blood pressure in both human and animal models [21,22]. However, it is not known if dietary salt affects memory and learning in mice carrying the APOE4 allele. The goal of the present study was to determine if there is a direct link between spatial learning, memory, high dietary salt, and expression of the APOE4 allele. We hypothesized that a high-salt diet would cause impaired spatial learning and memory, and increase neuroinflammation in mice carrying the human APOE4 allele. Here, we demonstrate, for the first time, that a prolonged high-salt (4% NaCl) diet caused a significant decrease in spatial learning and memory in mice carrying the human APOE4 allele, while the APOE4 mice on a low-salt diet (0.1% NaCl) showed improvement in spatial learning and memory on the Barnes maze behavioral test.

Low dietary salt intake is associated with decreased thirst, while high dietary salt intake increases thirst. There has been conflicting data showing either no change or a decrease in urinary output in low dietary salt conditions. Some studies have demonstrated that changing dietary salt from normal to a low-salt diet does not change water intake but can decrease urine output both in humans [23] and in mice [24]. We observed a significant decrease in urine output without changes in water intake in both the APOE3 and APOE4 allele-carrying mice when changing the diet to a low-salt diet from a normal-salt diet. Further studies are required to determine the cause of changes in urinary volume due to salt intake. As expected, when dietary salt was increased to 4%, the urinary output increased in both animal groups. We also showed a significant difference in body weight between male and female animals in both the APOE3 and APOE4 allele-carrying mice, despite no significant differences in water and food intake between the animal groups. One limitation of our study is that we did not measure the serum and urinary sodium, chloride, and potassium in these animals.

Hypertension is one of the common disorders affecting more than one billion adults all over the world [25]. The highest prevalence (54%) is among non-Hispanic black adults and increases from about 60% in 45–64-year-olds to about 77% in those aged 65 and older. The majority of hypertensive patients above 65 years of age are salt-sensitive [26]. Several studies have demonstrated an increased risk of dementia and cognitive impairment in people with hypertension [27]. Hypertension is linked with cerebrovascular disease due to its effects on the intracranial vasculature, which include changes in microvasculature, loss of endothelial function, and a decrease in cerebral blood flow, causing regional hypoxia [28]. Magnetic resonance imaging techniques have shown that hypertension is associated with cerebral small vessel disease, microbleeds, white matter hyperintensities, basal ganglia perivascular spaces, lacunar strokes, and cerebral amyloid angiopathies [29]. High blood pressure has also been shown to be associated with several AD pathologies [30], including β-amyloid deposition, phosphorylation of tau, and intracellular tau tangles [31]. We, and others, have demonstrated that high dietary salt increases blood pressure [17,18,32] and expression of cytokines in aged mice [33]. Our data showed no significant differences in the memory parameters on TD4 following dietary changes. However, the data showed significantly decreased memory recall, as observed by the latency to the target hole in the APOE4 mice in the 72 h. probe. These data suggest that prolonged high dietary salt may cause memory loss at an early age in mice carrying the APOE4 allele. However, further studies are required to confirm these observations. In the present study, we demonstrate that high dietary salt decreased memory function in mice carrying the APOE4 allele as compared to animals carrying the normal APOE3 allele, suggesting a direct link between dietary salt, memory loss, and the APOE4 allele. It is also postulated that APOE4 contributes to increased microglial activation, which was observed by an increase in Iba1-positive microglial cells (Figure 5). The decrease in memory function in the APOE4 mice may be due to the rise in neuroinflammation within the hippocampal formation, an important area for learning and memory. The data presented in this study do not allow us to speculate on changes in blood pressure due to the lack of direct physiological outputs, such as blood pressure, urinary volume, and sodium and potassium clearance. Whether high dietary salt will have effects similar to hypertension on AD pathologies remains to be investigated.

## 5. Limitations

This study has several limitations. A key limitation of this study is the absence of direct blood pressure measurements. Although prior studies from our laboratory and others have shown that high dietary salt increases blood pressure, we cannot directly determine whether the observed cognitive changes are mediated by hemodynamic changes in this model. Second, neuroinflammation was assessed using Iba1 alone, without additional microglial activation markers or cytokine profiling. To better characterize the inflammatory response, future studies will include additional markers of microglial activation, such as CD68 and CD11b, as well as inflammatory cytokines (e.g., IL-1β, TNF-α). Third, only the Barnes maze was used to assess cognition, and additional behavioral assays would strengthen the interpretation of the observed memory phenotype. Finally, serum and urinary electrolyte measurements were not performed, which limits the physiological interpretation of the dietary interventions.

## 6. Conclusions

In summary, we show that high dietary salt increases urinary output in both APOE3 and APOE4 mice and impairs memory in APOE4 mice. In humans, many studies have shown the benefit of healthy diets, such as the Mediterranean diet or the Dietary Approach to Stop Hypertension diet, which are beneficial for better cognitive function [34,35]. Individuals with or without APOE3 and/or APOE4 alleles could benefit from a low-salt diet due to its benefits on cardiovascular and cerebral health.

## Data Availability

The raw data supporting the conclusions of this article will be made available by the authors on request.

## Supplementary Materials

The following supporting information can be downloaded at: https://www.mdpi.com/article/10.3390/neurosci7020043/s1, Figure S1: Effect of dietary salt on body weight and food intake in male and female APOE3 and APOE4 mice; Figure S2: Effect of dietary salt on water intake and urine output; Figure S3: Effect of dietary salt on latency to target hole on training days; Figure S4: Baseline Primary Latency (A); Primary errors (B); Distance traveled (C); and Total nose pokes on training day 4; Figure S5: Effect of dietary salt on latency to target hole in male and female mice on training days.

## Abbreviations

The following abbreviations are used in this manuscript:

APOE	Apolipoprotein Epsilon
AD	Alzheimer Disease
HDL	High-density lipoproteins
VLDL	Very low-density lipoproteins
CVD	Cardiovascular disease
CKD	Chronic kidney disease
Iba1	Ionized calcium binding adaptor molecule 1

## References

[1] Matsunaga A., Saito T. (2014). Apolipoprotein E mutations: A comparison between lipoprotein glomerulopathy and type III hyperlipoproteinemia. Clin. Exp. Nephrol..

[2] Greenow K., Pearce N.J., Ramji D.P. (2005). The key role of apolipoprotein E in atherosclerosis. J. Mol. Med..

[3] Suarez B.K., Schonfeld G. (1981). Characterization of apolipoprotein E (ApoE) apoprotein levels in the various ApoE phenotypes. J. Clin. Endocrinol. Metab..

[4] Han S., Xu Y., Gao M., Wang Y., Wang J., Liu Y., Min W., Zhang X. (2016). Serum apolipoprotein E concentration and polymorphism influence serum lipid levels in Chinese Shandong Han population. Medicine.

[5] Czaplińska M., Ćwiklińska A., Sakowicz-Burkiewicz M., Wieczorek E., Kuchta A., Kowalski R., Kortas-Stempak B., Dȩbska-Ślizień A., Jankowski M., Król E. (2019). Apolipoprotein E gene polymorphism and renal function are associated with apolipoprotein E concentration in patients with chronic kidney disease. Lipids Health Dis..

[6] Jackson R.J., Hyman B.T., Serrano-Pozo A. (2024). Multifaceted roles of APOE in Alzheimer disease. Nat. Rev. Neurol..

[7] Nguyen D., Dhanasekaran P., Nickel M., Nakatani R., Saito H., Phillips M.C., Lund-Katz S. (2010). Molecular basis for the differences in lipid and lipoprotein binding properties of human apolipoproteins E3 and E4. Biochemistry.

[8] MacKensen G.B., Swaminathan M., Ti L.K., Grocott H.P., Phillips-Bute B.G., Mathew J.P., Newman M.F., Milano C.A., Stafford-Smith M. (2004). Preliminary report on the interaction of apolipoprotein E polymorphism with aortic atherosclerosis and acute nephropathy after CABG. Ann. Thorac. Surg..

[9] Hunsberger H.C., Pinky P.D., Smith W., Suppiramaniam V., Reed M.N. (2019). The role of APOE4 in Alzheimer’s disease: Strategies for future therapeutic interventions. Neuronal Signal..

[10] Ferrari C., Lombardi G., Polito C., Lucidi G., Bagnoli S., Piaceri I., Nacmias B., Berti V., Rizzuto D., Fratiglioni L. (2018). Alzheimer’s Disease Progression: Factors Influencing Cognitive Decline. J. Alzheimers Dis..

[11] Serrano-Pozo A., Das S., Hyman B.T. (2021). APOE and Alzheimer’s disease: Advances in genetics; pathophysiology; therapeutic approaches. Lancet Neurol..

[12] Malone J.E., Elkasaby M.I., Lerner A.J. (2022). Effects of Hypertension on Alzheimer’s Disease and Related Disorders. Curr. Hypertens. Rep..

[13] Fuchs F.D., Whelton P.K. (2020). High Blood Pressure and Cardiovascular Disease. Hypertension.

[14] Stefaniak O., Dobrzyńska M., Drzymała-Czyż S., Przysławski J. (2022). Diet in the Prevention of Alzheimer’s Disease: Current Knowledge and Future Research Requirements. Nutrients.

[15] Van Vliet B.N., Montani J.P. (2008). The time course of salt-induced hypertension, and why it matters. Int. J. Obes..

[16] Bailey M.A., Dhaun N. (2024). Salt Sensitivity: Causes, Consequences, and Recent Advances. Hypertension.

[17] Nishimoto M., Griffin K.A., Wynne B.M., Fujita T. (2024). Salt-Sensitive Hypertension and the Kidney. Hypertension.

[18] Pushpakumar S., Ahmad A., Ketchem C.J., Jose P.A., Weinman E.J., Sen U., Lederer E.D., Khundmiri S.J. (2020). Sodium-hydrogen exchanger regulatory factor-1 (NHERF1) confers salt sensitivity in both male and female models of hypertension in aging. Life Sci..

[19] Sepulveda J., Luo N., Nelson M., Ng C.A.S., Rebeck G.W. (2022). Independent APOE4 knock-in mouse models display reduced brain APOE protein, altered neuroinflammation, and simplification of dendritic spines. J. Neurochem..

[20] Tripathi S., Sharma Y., Kumar D. (2024). Unraveling APOE4’s Role in Alzheimer’s Disease: Pathologies and Therapeutic Strategies. Curr. Protein Pept. Sci..

[21] Dobrian A.D., Schriver S.D., Lynch T., Prewitt R.L. (2003). Effect of salt on hypertension and oxidative stress in a rat model of diet-induced obesity. Am. J. Physiol.-Ren. Physiol..

[22] Boegehold M.A. (2013). The effect of high salt intake on endothelial function: Reduced vascular nitric oxide in the absence of hypertension. J. Vasc. Res..

[23] Juraschek S.P., Miller E.R., Chang A.R., Anderson C.A.M., Hall J.E., Appel L.J. (2020). Effects of Sodium Reduction on Energy, Metabolism, Weight, Thirst, and Urine Volume: Results From the DASH (Dietary Approaches to Stop Hypertension)-Sodium Trial. Hypertension.

[24] Veress A.T., Chong C.K., Field L.J., Sonnenberg H. (1995). Blood pressure and fluid-electrolyte balance in ANF-transgenic mice on high- and low-salt diets. Am. J. Physiol. Integr. Comp. Physiol..

[25] First WHO Report Details Devastating Impact of Hypertension and Ways to Stop It. https://www.who.int/news/item/19-09-2023-first-who-report-details-devastating-impact-of-hypertension-and-ways-to-stop-it.

[26] Balafa O., Kalaitzidis R.G. (2020). Salt sensitivity and hypertension. J. Hum. Hypertens..

[27] Ungvari Z., Toth P., Tarantini S., Prodan C.I., Sorond F., Merkely B., Csiszar A. (2021). Hypertension-induced cognitive impairment: From pathophysiology to public health. Nat. Rev. Nephrol..

[28] Amier R.P., Marcks N., Hooghiemstra A.M., Nijveldt R., van Buchem M.A., de Roos A., Biessels G.J., Kappelle L.J., van Oostenbrugge R.J., van der Geest R.J. (2021). Hypertensive Exposure Markers by MRI in Relation to Cerebral Small Vessel Disease and Cognitive Impairment. JACC Cardiovasc. Imaging.

[29] Zhao L., Lee A., Fan Y.H., Mok V.C.T., Shi L. (2021). Magnetic resonance imaging manifestations of cerebral small vessel disease: Automated quantification and clinical application. Chin. Med. J..

[30] Marfany A., Sierra C., Camafort M., Domenech M., Coca A. (2018). High blood pressure, Alzheimer disease and antihypertensive treatment. Panminerva Med..

[31] Shih Y.H., Wu S.Y., Yu M., Huang S.H., Lee C.W., Jiang M.J., Lin P.Y., Yang T.T., Kuo Y.M. (2018). Hypertension accelerates Alzheimer’s disease-related pathologies in pigs and 3xTg mice. Front. Aging Neurosci..

[32] Nishimoto M., Fujita T. (2015). Renal mechanisms of salt-sensitive hypertension: Contribution of two steroid receptor-associated pathways. Am. J. Physiol.-Ren. Physiol..

[33] Jain A., Jung H.J., Aubee J., O’Neil J.N., Muhammad L.A., Khan S., Thompson K., Fluitt M.B., Lee D.L., Klinge C.M. (2024). Role of NHERF1 in MicroRNA Landscape Changes in Aging Mouse Kidneys. Biomolecules.

[34] Aridi Y.S., Walker J.L., Wright O.R.L. (2017). The Association between the Mediterranean Dietary Pattern and Cognitive Health: A Systematic Review. Nutrients.

[35] Berendsen A.A.M., van de Rest O., Feskens E.J.M., de Groot L.C.P.G.M., Kang J.H., Grodstein F., Grodstein F. (2017). The Dietary Approaches to Stop Hypertension Diet, Cognitive Function, and Cognitive Decline in American Older Women. J. Am. Med. Dir. Assoc..

