# Supplementary material for: Effect of High-Salt Diet on Memory and Behavior in Mice Expressing Human Apolipoprotein Epsilon-4 (APOE4) Allele
Source: NeuroSci. 2026 Apr 7;7(2):43. doi: 10.3390/neurosci7020043 (PMC13119126; doi:10.3390/neurosci7020043)

Supplementary Figure S1: Effect of Dietary Salt on Body Weight and Food Intake in Male and Female APOE3 and APOE4 mice

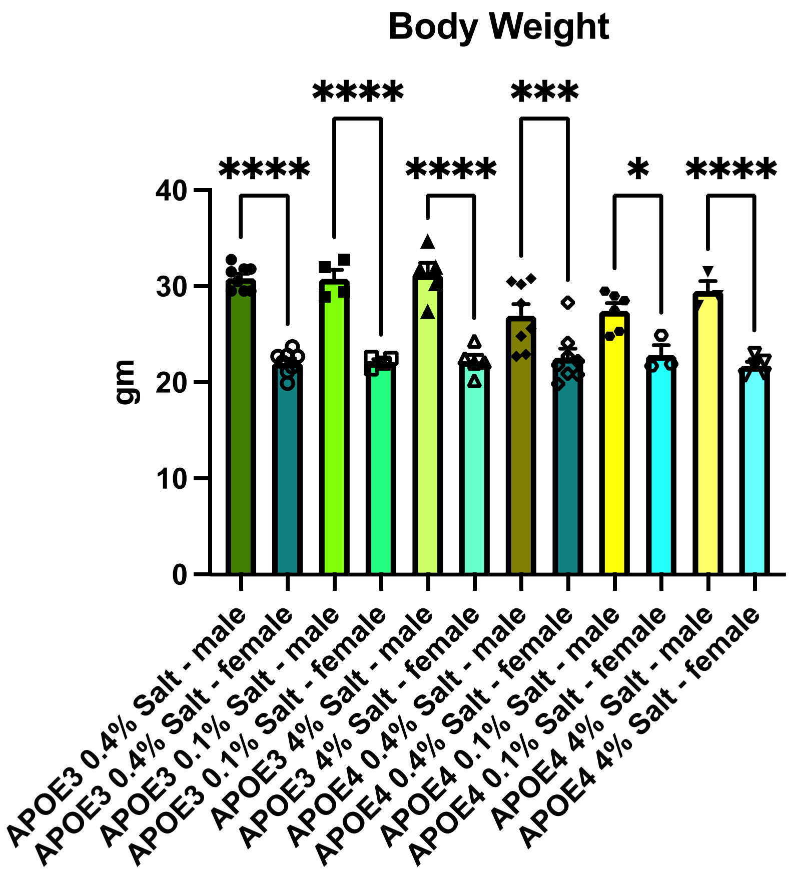

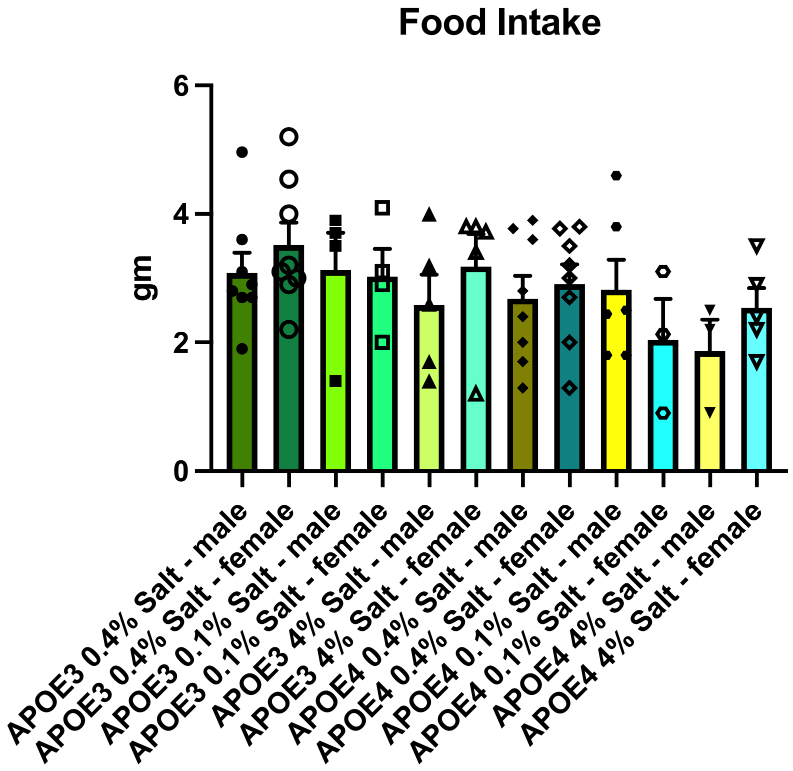

Supplementary Figure S2: Effect of dietary salt on water intake and urine output

(A)

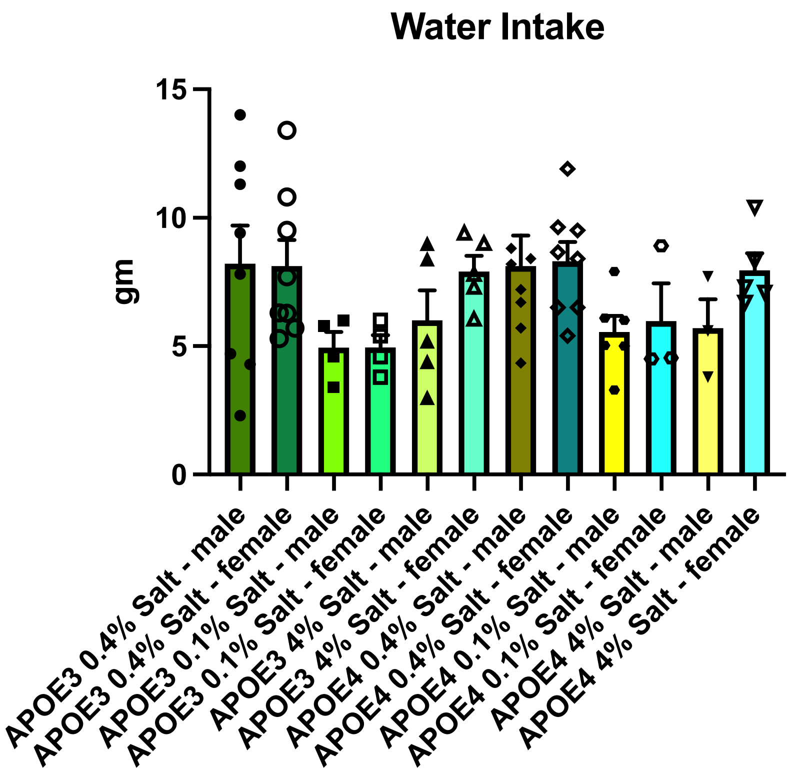

(B)

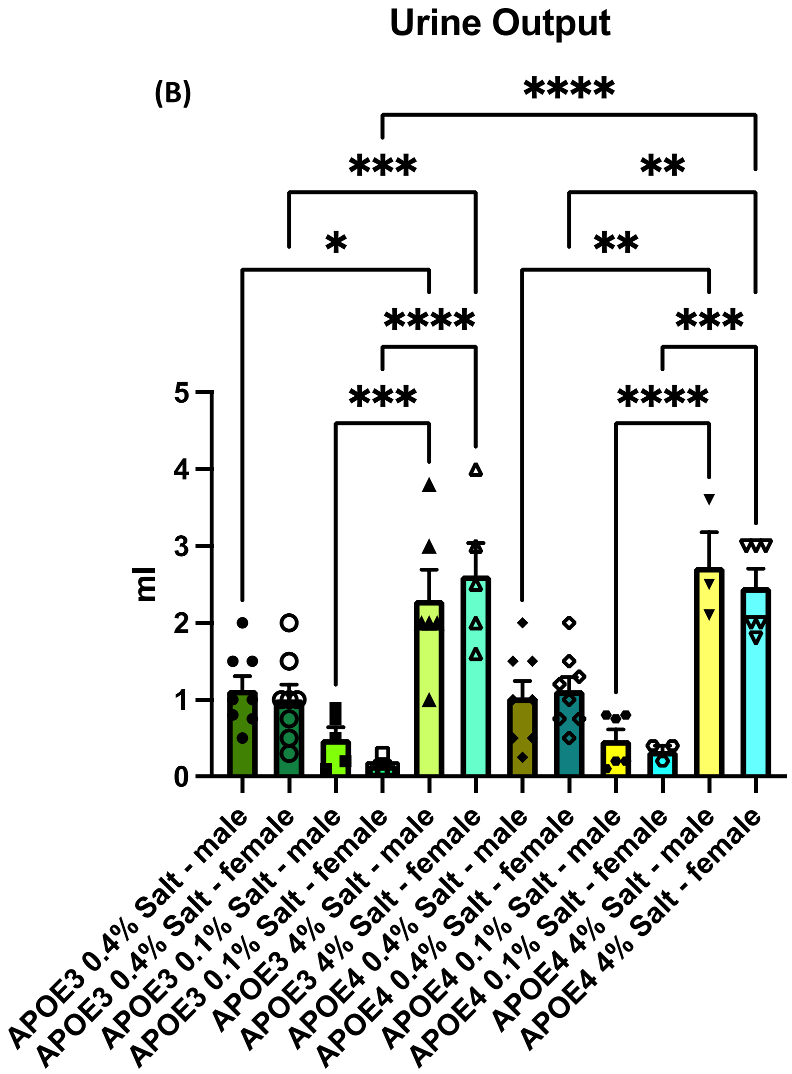

Supplementary Figure S3: Effect of dietary salt on Latency to target hole on training days

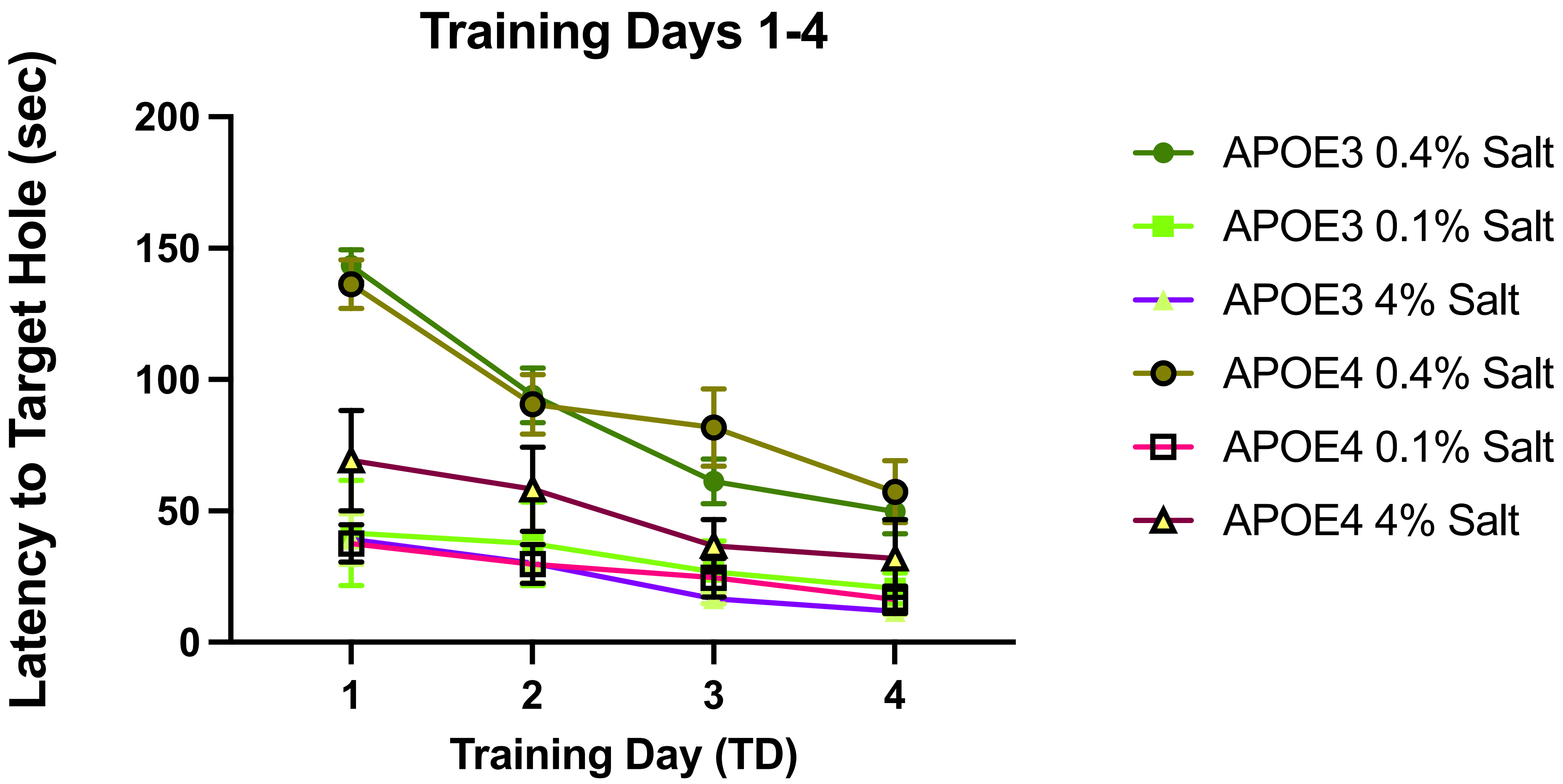

Supplementary Figure S4: Baseline Primary Latency (A); Primary errors (B); Distance traveled (C); and Total nose pokes on training day 4

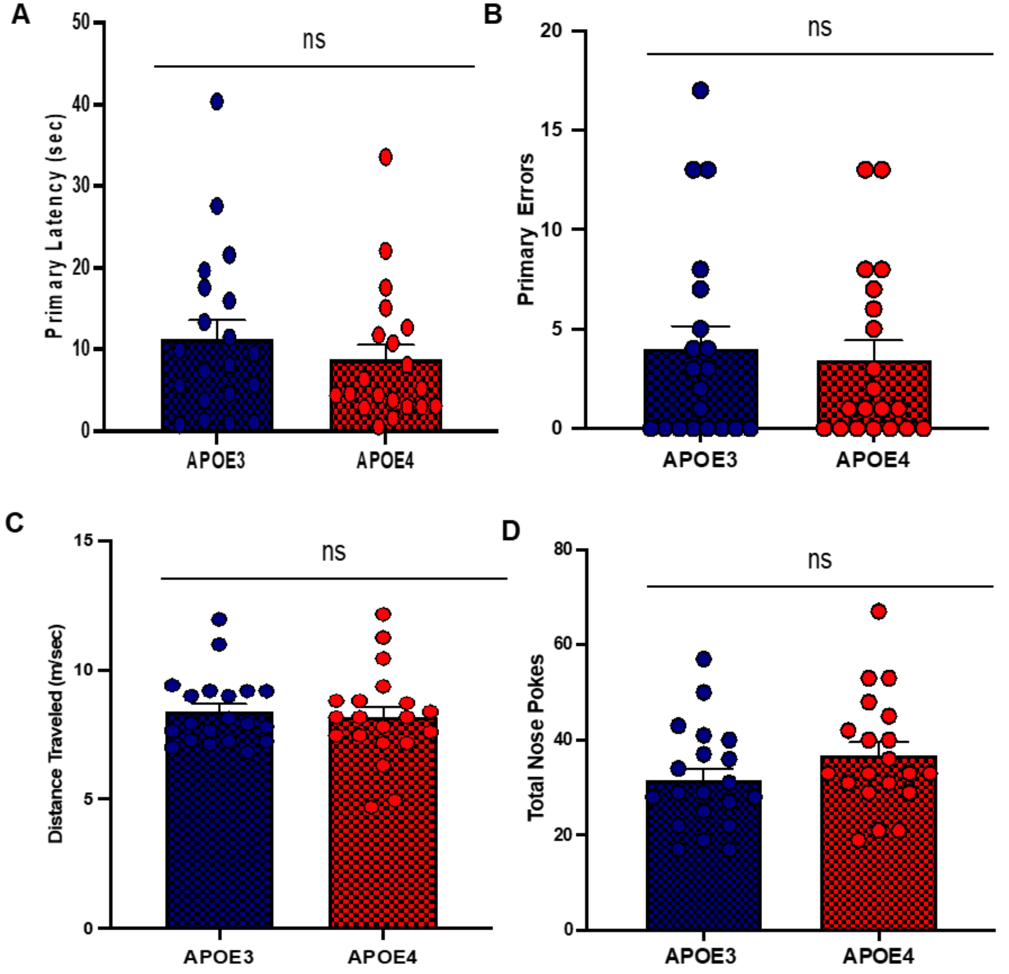

Supplementary Figure S5: Effect of dietary salt on Latency to target hole in male and female mice on training days

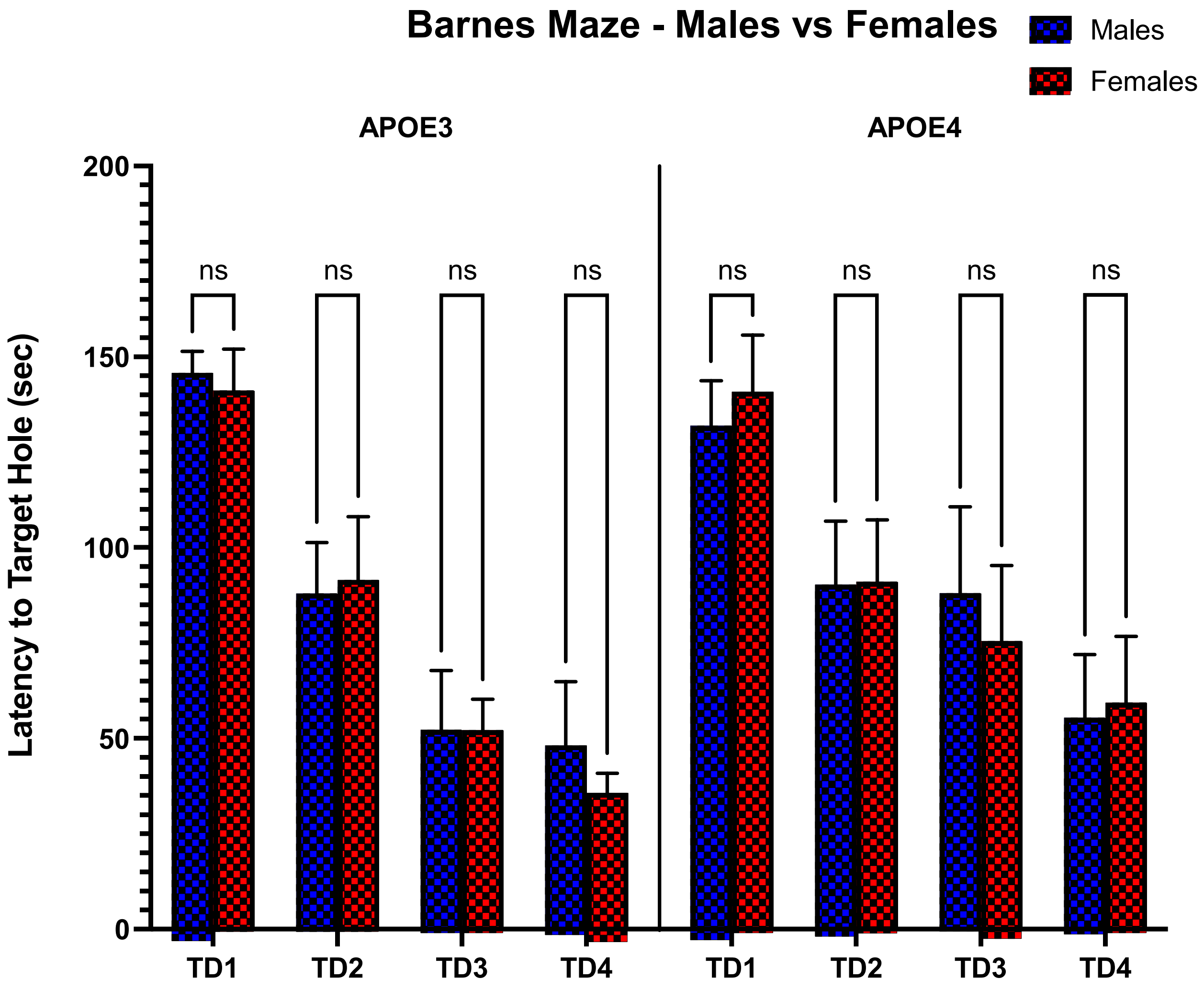

Supplement: Supplementary file 1 [file neurosci-07-00043-s001.zip › neurosci-4167292-supplementary.pdf]
